# The Effect of Grazing on Central Anatolian Steppe Vegetation: A Modeling Approach Using Functional Traits

**DOI:** 10.1002/ece3.70499

**Published:** 2024-11-03

**Authors:** Anıl Bahar, Çağatay Tavşanoğlu

**Affiliations:** ^1^ Institute of Science Hacettepe University Ankara Türkiye; ^2^ Division of Ecology, Department of Biology Hacettepe University Ankara Türkiye

**Keywords:** Anatolian steppes, disturbance, grazing regimes, modeling, plant functional groups, temperate grasslands, vegetation dynamics

## Abstract

Grazing is a major ecological driver that influences vegetation dynamics globally. We investigated the long‐term effects of different grazing regimes on the vegetation structure of the Central Anatolian steppes, a region characterized by its unique convergence of biogeographical influences and historical land use. We employed the spatially explicit FATELAND model to simulate vegetation dynamics over a 50‐year period under three distinct grazing scenarios: no grazing, moderate grazing, and overgrazing. Our simulations incorporated a range of plant functional traits to predict changes across five different vegetation types in Central Anatolia, including woodland steppes and treeless steppes. The simulations revealed that moderate grazing supports the diversity and abundance of various plant functional groups, excluding resprouter trees, which flourish under no grazing conditions. In contrast, overgrazing leads to significant reductions in the abundance of perennial forbs and both spiny and non‐spiny subshrubs, often resulting in a shift toward grassland dominated by resprouter gramineae or an annual herb‐dominated grassland, depending on the initial abundance of gramineae. Our findings highlight the critical role of grazing management in maintaining biodiversity and ecological stability in steppe ecosystems. While moderate grazing can enhance plant functional group diversity, overgrazing significantly threatens the ecological integrity of the Central Anatolian steppes. In conclusion, our modeling approach reveals that the grazing regime is a major driver in shaping the vegetation structure of Central Anatolian steppes. Grazing management strategies that are adjusted to the ecological characteristics and historical context of specific regions are required to prevent degradation and promote sustainable grassland vegetation.

## Graphical abstract

We studied the long‐term effects of various grazing regimes on the vegetation structure of the Central Anatolian steppes using the FATELAND model to simulate changes over 50 years. Our simulations, which incorporated different plant functional traits, show that moderate grazing supports biodiversity, whereas overgrazing leads to a significant decrease in several plant groups. These results emphasize the crucial role of grazing management in maintaining the ecological stability of steppe ecosystems. Effective grazing management strategies adjusted to regional ecological and historical contexts are essential to prevent degradation and promote sustainable vegetation.

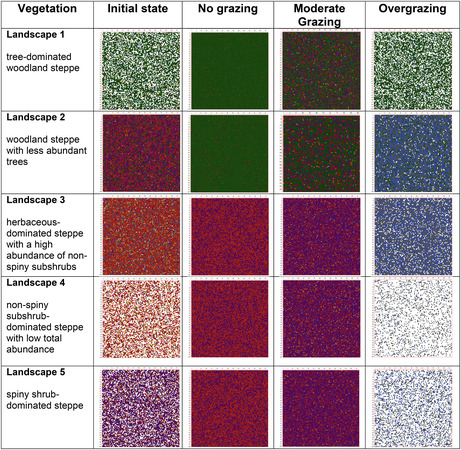

## Özet

Otlatma, küresel olarak vejetasyon dinamiklerini etkileyen önemli ekolojik bir faktördür. Bu çalışmada, geçmişteki arazi kullanımının belirgin biçimde öne çıktığı ve biyocoğrafi olarak eşsiz bir bölgede yer alan İç Anadolu bozkırlarında, farklı otlatma rejimlerinin uzun vadeli etkileri incelenmiştir. Vejetasyon dinamiklerini 50 yıllık bir süre boyunca üç farklı otlatma senaryosu altında simüle etmek için mekânsal özellikli FATELAND modelini kullandık: otlatma yok, orta düzeyde otlatma ve aşırı otlatma. Simülasyonlarımız, ağaçlı ve ağaçsız bozkırlar dahil, İç Anadolu'daki beş farklı vejetasyon tipinde, değişiklikleri tahmin etmek için çeşitli bitki fonksiyonel karakterlerini içermektedir. Simülasyonlar, orta düzeyde otlatmanın bitki fonksiyonel grubu çeşitliliğini ve bolluğunu desteklediğini, ancak yeniden sürgün verebilen ağaçların yalnızca otlatma olmayan koşullarda arttığını göstermiştir. Buna karşılık, aşırı otlatma, çok yıllık otsu bitkilerin ve dikenli/dikensiz yarı çalıların bolluğunda önemli seviyede azalmalara yol açmış ve genellikle, başlangıçtaki gramineae bolluğuna bağlı olarak, yeniden sürgün verebilen gramineae'lerin hâkim olduğu çayırlara veya tek yıllık otlarla kaplı çayırlara dönüşüme neden olmuştur. Bulgularımız, bozkır ekosistemlerinde biyoçeşitliliği ve ekolojik kararlılığı sürdürmede otlatma yönetiminin kritik bir rolü olduğunu göstermektedir. Orta düzeyde otlatma bitki fonksiyonel grup çeşitliliğini artırabilirken, aşırı otlatma İç Anadolu bozkırlarının ekolojik bütünlüğünü ciddi şekilde tehdit etmektedir. Sonuç olarak, modelleme yaklaşımımız, otlatma rejiminin İç Anadolu bozkırlarının vejetasyon yapısını şekillendirmede başlıca bir faktör olduğunu ortaya koymaktadır. Belirli bölgelerin ekolojik özelliklerine ve tarihi bağlamına uygun olarak tasarlanmış otlatma yönetimi stratejileri, arazi bozulmasını önlemek ve sürdürülebilir bir çayır vejetasyonunu teşvik etmek için gereklidir.

## 1 Introduction

Grazing has the ability to significantly alter ecosystems on a global scale (Asner et al. 2004; Zhang et al. 2023). Many open ecosystems, such as grasslands are maintained by disturbances, including vertebrate herbivory (Evans et al. 2015; Dantas et al. 2016; Bond 2019). The intensity of grazing markedly influences the abundance of herbaceous species in savanna and grassland ecosystems (Hayes and Holl 2003; Gebremedhn et al. 2023) and can alter the spatial heterogeneity of vegetation (Adler, Raff, and Lauenroth 2001). Moderate and low‐intensity grazing play a crucial role in sustaining high levels of biodiversity within grassland ecosystems (Isselstein et al. 2007; Török et al. 2016; Joubert, Pryke, and Samways 2017; Wolański et al. 2021; Davies et al. 2024). Overgrazing, on the other hand, leads to a significant decline in grassland biodiversity (Wesche et al. 2016; Rahmanian et al. 2019; Munkhzul et al. 2021; Zhang, Wang, and Niu 2021). Many studies on the impact of grazing on grasslands have shown that low‐intensity grazing has a positive effect, while overgrazing negatively impacts grassland biodiversity (Metera et al. 2010). For example, in Mongolian temperate grasslands, increasing grazing intensity negatively affects plant cover and aboveground biomass, with high‐intensity grazing leading to a decline in tall grasses and an increase in short grasses (Zainelabdeen et al. 2020). European grasslands also show this trend: the type of grazer affects biodiversity and species composition; cattle grazing, compared to sheep grazing, promotes more trait‐rich vegetation with higher forb cover (Tóth et al. 2018). A long‐term field experiment in North American semi‐arid grasslands demonstrated that long‐term grazing causes slow, continuous changes in plant communities without inducing alternative stable states, emphasizing the importance of understanding phase shifts rather than focusing solely on thresholds between states (Porensky et al. 2016). In savanna grasslands, heavy cattle grazing can increase tree density by reducing grass biomass and creating more open sites for tree seedling establishment, which may eventually lead to the cessation of grazing in woody‐encroached grasslands (Mogashoa, Dlamini, and Gxasheka 2021).

Indeed, grassland vegetation undergoes dynamic changes over time in response to disturbances such as herbivory and fire (Van Langevelde et al. 2003; Sankaran, Ratnam, and Hanan 2004; Baudena et al. 2015; Dantas et al. 2016; Bond 2019). As a result, it often coexists with or transitions into alternative stable states with woodlands (Beisner, Haydon, and Cuddington 2003; Pausas and Bond 2020). Herbivory not only promotes grass establishment but also regulates tree cover in savannas (Van Langevelde et al. 2003; Bond 2019). Grazing is one of the primary drivers of state transition in grasslands (Twidwell, Allred, and Fuhlendorf 2013), although these changes takes more than a decade to occur (Milchunas 2011; Bestelmeyer et al. 2013). In many other parts of the world, however, the effect of grazing on grassland vegetation also depends on vegetation type and local climate (Munkhzul et al. 2021).

The temperate grasslands of Central Anatolia are renowned for their extraordinary biodiversity, primarily due to the unique convergence of plant species from both the Irano‐Turanian and Mediterranean phytogeographic regions (Ekim and Güner 2000; Kurt, Nilhan, and Ketenoglu 2006). The region has a long‐standing history of pastoralism, shaping its landscape for nearly 10,000 years (Hammer and Arbuckle 2017; Middleton 2018). Archeological evidence suggests the earliest domestication of herbivores in the region around 9000 cal BC, with earlier evidence from the Eastern Mediterranean dating back to 12,000 cal BC (Payne 1985; Zeder 2008; Middleton 2018). Additionally, aurochs (*Bos primigenius*) and mouflon (*Ovis orientalis*) existed in Anatolia during the Neolithic ages (Perkins and Daly 1968). The domestication timeline reveals that sheep and goats were domesticated before cattle, with domestic pigs being domesticated much later than in surrounding regions (Arbuckle 2013; Peters et al. 2013). Domestic grazing, a form of herbivory regulated by human intervention, involves the consumption of vegetation by domesticated mammalian species (Metera et al. 2010; Rosenthal, Schrautzer, and Eichberg 2012). Herbivory, on the other hand, involves the consumption of plants by a mix of domestic and wild animals, ranging from insects and rodents to large mammals. Currently, domestic grazing, predominantly by cattle, sheep, and goats, is the main form of mammal herbivory in Central Anatolian steppes (Fırıncıoğlu, Seefeldt, and Şahin 2007; Tavşanoğlu 2017). Over the last 50 years in Central Anatolia, intensified agricultural activities led to the conversion of grasslands to croplands, driving overgrazing in many remaining rangelands (Fırıncıoğlu, Seefeldt, and Şahin 2007; Ambarlı et al. 2016). Consequently, overgrazing has been a major driver of habitat degradation in many parts of Central Anatolia (Kürschner and Parolly 2012; Koc, Schacht, and Erkovan 2015; Ambarlı et al. 2016; Gökbulak et al. 2018).

It is widely recognized that overgrazing is a primary factor in vegetation degradation in Central Anatolia (Ünal and Fırıncıoğlu 2007; Gökbulak et al. 2018). In his region, grazing significantly reduces the cover of forbs and grasses (Fırıncıoğlu et al. 2009, 2010), with forbs experiencing the most substantial negative impact (Fırıncıoğlu, Seefeldt, and Şahin 2007). However, the cover of cushion‐type subshrubs appears similar in both grazed and ungrazed areas (Fırıncıoğlu et al. 2010). Certain subshrub species, such as *Thymus* spp., have shown increased cover in grazed areas (Fırıncıoğlu et al. 2009). Such experiments suggest that plant cover is more robust in ungrazed areas than in grazed ones, plant diversity does not increase with grazing, and grasslands are transitioned to subshrub‐dominated sites following extensive overgrazing in Central Anatolia (Fırıncıoğlu, Seefeldt, and Şahin 2007; Fırıncıoğlu et al. 2010). On the other hand, an experiment that included artificial disturbances to vegetation and soil suggests that Central Anatolian vegetation has resilience to disturbance, at least on a small scale (Özüdoğru, Özüdoğru, and Tavşanoğlu 2021). The diversity of belowground organs in herbaceous plants of Anatolian steppes (Ülgen and Tavşanoğlu 2024) also highlights the resilience of this vegetation to various disturbances. Indeed, many species resprout after being clipped at ground level (Tavşanoğlu et al., unpublished data). In addition to grazing, seeds of Central Anatolian steppe plants can resist low‐intensity heat shocks, suggesting that these plants are also resilient to surface fire regimes (Tavşanoğlu, Çatav, and Özüdoğru 2015) which were frequent during the early‐ to mid‐Holocene (Turner et al. 2010). Although there are studies providing a general overview of the effects of grazing on Central Anatolian vegetation (Gintzburger, Le Houérou, and Saïdi 2006; Ambarlı et al. 2016; Tavşanoğlu 2017; Gökbulak et al. 2018) and a few studies mentioned above that provide experimental data from controlled experiments (Fırıncıoğlu, Seefeldt, and Şahin 2007; Fırıncıoğlu et al. 2009, 2010; Özüdoğru, Özüdoğru, and Tavşanoğlu 2021), the absence of long‐term studies hinders our ability to predict the dynamics of grazing–vegetation relationships in the steppe vegetation of Central Anatolia under global environmental changes. Although modeling studies may enhance our knowledge of long‐term dynamics in such cases, no modeling study has yet been conducted to specifically examine the impact of grazing on shaping the steppe vegetation of Central Anatolia. Considering the current acceleration of agricultural land abandonment and changes in domestic grazing regimes (Tavşanoğlu 2017), unraveling long‐term vegetation dynamics is crucial to fully understand the post‐abandonment recovery processes, conservation, and management of Anatolian steppe vegetation in the near future. Modeling the long‐term vegetation dynamics of the understudied Central Anatolian vegetation in response to various grazing regimes would also enhance our understanding of how grazing shapes temperate grassland ecosystems.

Vegetation ecology fundamentally revolves around patterns and processes that vary significantly across spatial and temporal scales (Wiegleb 1989). A pivotal aspect of this field is the use of disturbance‐based vegetation models in which are instrumental in testing hypotheses about vegetation changes under disturbances of various frequency and intensity (Adler, Raff, and Lauenroth 2001; Pausas 2006; Seidl et al. 2011). In disturbance‐prone environments, models that incorporate both disturbance and response mechanisms become essential for thoroughly understanding vegetation mechanics (Lavorel et al. 1997; Millington et al. 2009). Disturbance‐based models offer advantages over dynamic global vegetation models, gap models, and resource‐based models by effectively simulating the direct impacts of disturbances and ongoing dynamics in ecosystems (He and Mladenoff 1999; Guiot and Cramer 2016; Baudena et al. 2020; Holdo and Nippert 2023). With their detailed focus on plant functional traits and responses to disturbances, these models provide more realistic predictions, especially in ecosystems frequently exposed to disturbance, compared to dynamic global vegetation models and gap models (Pausas 1999; Bugmann 2001; Risch, Heiri, and Bugmann 2005). For instance, the FATELAND model, which has been rigorously tested in fire‐prone Mediterranean ecosystems (Pausas 2006; Pausas, Lloret, and Vila 2006; Pausas and Lloret 2007; Bahar 2018), effectively represents vegetation mechanics in these environments (Millington et al. 2009). Such models allow us to predict long‐term vegetation dynamics under various disturbance regimes, including grazing intensity and provide valuable insights for future ecological forecasting for conservation and management.

In this study, we aim to unravel the long‐term dynamics of Central Anatolian steppes using the disturbance‐based modeling approach utilizing plant functional traits. Given that grazing is the primary disturbance factor in the Central Anatolian steppe ecosystem, and considering the variety of vegetation types in the region, our study included five distinct vegetation types subjected to varying grazing regimes. The overarching objective is to comprehensively understand the influence of grazing on the structural development of vegetation. Additionally, our study aims to examine the possible occurrence of different vegetation states under the differential pressures imposed by various grazing regimes. Based on our knowledge from other grazing‐meditated grassland ecosystems, we hypothesized that moderate grazing simulation would support the stability of the studied vegetation by maintaining growth form diversity, while simulations of no grazing and overgrazing would have negative effects, potentially leading to vegetation state changes in the latter scenarios. We also expected different plant functional groups to respond differently to various grazing regime simulations. To test these hypotheses, we used the spatially explicit FATELAND model to predict 50 years of vegetation dynamics under alternative grazing regimes by examining the dynamics of plant functional groups with varying traits related to growth, reproduction, and response to grazing.

## 2 Methods

### 2.1 Study Area

**FIGURE 1:**
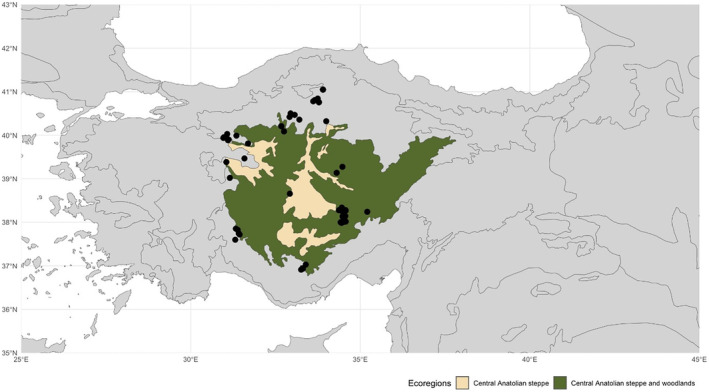
The map of the studied ecoregions; Central Anatolian steppes (marked in yellow) and Central Anatolian woodlands and steppes (marked in green), and the locations of vegetation data gathered from phytosociological studies used to create initial landscapes for the model. The ecoregion map is based on Olson et al. (2001).

The study area is located in Central Anatolia, Türkiye, within the Irano‐Anatolian biodiversity hotspot (Mittermeier et al. 2005; Şekercioğlu et al. 2011), and covers two ecoregions, namely Central Anatolian Steppes and Central Anatolian Woodlands and Steppes (Figure 1). Most of Central Anatolia is a plain with an elevation range between 750 and 1250 m, but there are also several volcanic mountains exceeding 2000 m. This region has a semi‐arid climate characterized by cold winters and warm, dry summers, with annual precipitation ranging from 300 to 650 mm, and annual average temperature varies between 7°C and 13°C across the region (Bayer‐Altın 2023). The diversity in bedrock types includes volcanic (andesite, basalt, tuff, and agglomerate), rhyolite, ignimbrite, radiolarite, flysch, marly, serpentine, calcareous, gypsum, limestone, and rocky slopes. Correspondingly, various soil types occur on these geological materials. This spatial variation in bedrock and soil types significantly influences the diversity of vegetation types in the area. The vegetation types in the Central Anatolian steppe are primarily categorized as grasslands dominated by grassy plants, dry steppes dominated by chamaephyte shrubs, forest steppes, and saline steppes (Kurt, Nilhan, and Ketenoglu 2006; Kürschner and Parolly 2012). The diversity of different growth forms, long‐standing disturbance effect, and climatic variability also create a trait diversity among plants of Anatolian grasslands, including the Central Anatolian steppe (Ülgen 2019; Ülgen and Tavşanoğlu 2024).

### 2.2 The Model and Simulation Scenarios

We employed the spatially explicit, grid‐based FATELAND model (Pausas and Ramos 2006; accessible at https://www.uv.es/jgpausas/lass.htm) to simulate long‐term vegetation dynamics in our studied ecosystem. FATELAND incorporates disturbances and responses along with competition between plant species or functional groups. The model operates on annual time steps, simulating plant cohorts transitioning through discrete stages: propagules, seedlings, immature, and mature plants. Each grid cell can contain multiple species (Pausas and Ramos 2006), with abundance measured on a qualitative scale, categorized as absent, low, medium, or high within each cell. Survival within the model is represented by a matrix that accounts for survival probabilities across various life stages under different resource levels (low, medium, and high). The model also incorporates the effect of stratum (or height) on species dynamics, where taller plants have a competitive advantage in accessing resources, influencing their survival rates and overall abundance (Pausas and Ramos 2006). Survival is represented by a matrix of nine elements, where each element indicates whether survival occurs (1 for yes and 0 for no) across three life stages—germinants (seedlings), immatures, and matures—evaluated under three different resource levels: low, medium, and high. The default matrix assumes universal survival across all stages and resource levels. FATELAND is primarily a deterministic model, with the exception of its dispersal module, which introduces stochastic elements (Pausas and Ramos 2006). These mechanics allow the model to capture the complexity of plant community dynamics over time, reflecting the impact of resource availability and disturbances on population structure and distribution across the landscape.

We created three grazing regime scenarios: grazing exclusion (no grazing), moderate‐intensity grazing, and overgrazing. Disturbance events were arranged to occur annually. The “no grazing” scenario assumes the exclusion of grazing activity from the landscape for the entire simulation period. The moderate‐intensity grazing scenario represents a sustainable grazing regime, operating within the carrying capacity of rangelands. In contrast, the overgrazing scenario simulates heavy pressure on the vegetation, with livestock density approximately four times higher than the carrying capacity, reflecting conditions observed in many parts of Central Anatolia (Gintzburger, Le Houérou, and Saïdi 2006). Each scenario was run over a 50‐year period to capture both intermediate‐ and long‐term dynamics in the vegetation.

### 2.3 Creation of Initial Landscapes in the Model

To assess distinct initial landscape structures for modeling purposes, we created a dataset containing abundance data for plant species from phytosociological studies conducted in the region. The dataset comprises studies conducted in steppe and woodland‐steppe vegetation in Central Anatolia and its surroundings from 1961 to 2020 (Figure 1). These studies cover various vegetation types (i.e., alliances) commonly found in Central Anatolia and reflect the abundance and cover of plant species in the region. Additionally, the dataset includes family, genus and species names, growth forms, locality names, and coordinates where each study was conducted, and the reference (or cited original reference) for each study (Appensix S2). As the cover/abundance of plant species was measured in the field by following the Braun‐Blanquet method (Braun‐Blanquet 1932) in these studies, we converted these data to percentages for model use. In total, data from 668 relevés, 58 alliances, and 13 phytosociological studies across the Central Anatolian plain were compiled to create this dataset (Figure 1, Appendix S2).

We then transformed the species‐level abundance data into a new dataset by combining individual species trait data based on growth form, using plant trait databases such as TRY (Kattge et al. 2020) and BROT (Tavşanoğlu and Pausas 2018), as well as the published flora of Türkiye (Davis 1965–1985) and online flora sources such as World Flora Online (Borsch et al. 2020), TÜBİVES (Bakış, Babaç, and Uslu 2011), and numerous other online flora and herbarium websites (Table S1). This data compilation approach allowed for a detailed categorization of species according to their specific growth forms and traits. As a result, we identified five growth forms: tree, subshrub, perennial forb, perennial gramineae, and annual herb. We distinguished between perennial forbs and gramineae due to their known differential response to grazing (Fulbright et al. 2021; Gebremedhn et al. 2023). Furthermore, we divided the subshrub category into non‐spinous and spiny subshrubs by including the spinescence trait, as spinescence offers varying degrees of resistance to grazing. This categorization was based on the presence of spines, thorns, or prickles on the leaves or stems of subshrubs (based on Davis 1965–1985; Ülgen 2019; Kattge et al. 2020), placing them in the spiny subshrubs category. Note that trees in our model represent resprouting species such as oaks (*Quercus* spp.) and *Pyrus elaeagnifolia* as characterized by Central Anatolian woodland steppe (Kenar and Kikvidze 2019).

We determined the plant abundances in each functional group for each relevé by utilizing the maximum values of these functional groups' abundances (Appendix S3). Based on the abundance of growth forms in each type of vegetation, we performed a cluster analysis to consolidate 58 vegetation alliances into distinct vegetation types. For this, we employed the Elbow method, a technique used to determine the optimal number of clusters in the k‐means clustering algorithm (Figure S1; Jain 2010; Shi et al. 2021). This analysis resulted in the classification of all vegetation alliances under five distinct vegetation types: (1) tree‐dominated woodland steppe (hereafter “Landscape 1”), (2) woodland steppe with less abundant trees (hereafter, “Landscape 2”), (3) herbaceous‐dominated steppe with a high abundance of non‐spiny subshrubs (hereafter, “Landscape 3”), (4) non‐spiny subshrub‐dominated steppe with low total abundance (hereafter, “Landscape 4”), and (5) spiny shrub‐dominated steppe (hereafter, “Landscape 5”). Although both Landscape 1 and Landscape 2 can be classified as woodland steppe, the main difference between these two vegetation types was the total abundance of trees, with mean tree abundance being approximately as 50% in Landscape 1% and 10% in Landscape 2.

Finally, we created an initial landscape consisting of 10,000 cells (100 × 100 cells) in the FATELAND model for each landscape representing different vegetation types. We assumed that each cell measures 10 m × 10 m; therefore, each landscape covers a total area of 1000 m × 1000 m.

### 2.4 Traits and Response to Grazing

We used several functional traits to inform about the life history of each functional group while simulating vegetation dynamics in the FATELAND model (Tables 1, 2, 3). In the model, most traits and response parameters to disturbances were included as semiquantitative data (e.g., low‐medium‐high) or Boolean data (no/yes), while some quantitative traits were also presented. The model's reliance on the functional traits of various species or functional groups is informed by plant trait databases and field experience in the Central Anatolian steppes. Priority is given to the most abundant species in these databases, with traits and parameters detailed in Table 1. We randomly distributed all plants (at seed, immature, and mature stages) across the landscapes to establish their initial abundance. The FATELAND model initiates its simulation by defining the lifespan of functional groups through mature age and maximum age traits, thereby establishing their long‐term life histories (Tables 1 and 3). Seed dispersal distance and rate are determined based on their dispersal capabilities (Tables 1 and 3), which are crucial for incorporating spatial dynamics. The seed germination process is characterized by the fecundity trait, while the transformation of these seeds into immature individuals is governed by the germination trait (Tables 1 and 2). Thus, seed germination and the early development stages set the stage for how functional traits influence vegetation dynamics. Once seeds transform into immature individuals, the FATELAND model further examines how these growing plants interact with their environment. This progression from seed to mature plant is critical as it underscores the transition phases that directly impact plant survival and distribution (Pausas and Ramos 2006). Plant height is another crucial trait in the model that significantly influences the grazing response of functional groups. Taller growth forms, such as trees, have more resistance to grazing, particularly after they grow tall enough to escape the grazing zone. Therefore, the grazing responses of functional groups vary based on their maturity (immature versus mature life stages; Tables 1 and 3). This distinction, determined by the designated age for each group, indicates that mature individuals tend to be more resistant to grazing, while their immature counterparts are often more susceptible. Similarly, larger (or adult) plants have an advantage in resource acquisition, both belowground (root capacity) and aboveground (sunlight capture), compared to smaller (or immature) ones. This results in larger and adult plants having a higher resilience capacity to grazing than smaller or younger individuals (Tables 1 and 3). Consequently, both age class and height are represented in the disturbance (i.e., grazing) response of each functional group through the parameters “age limit,” “killresp,” and “respage” (Tables 1 and 3). These parameters and traits help model the differential impact of grazing on plants based on their size and maturity, reflecting more complex vegetation dynamics under various grazing pressures.

**TABLE 1:** Parameters and traits used in the FATELAND model (*sensu* Pausas and Ramos 2006). The main parameters besides disturbance‐ and germination‐related ones, along with their descriptions and categories, are given. Traits without categories are quantitative ones.

Parameters	Description	Categories
Main parameters		
Maxab	The maximum number of species in each cell in the landscape (1: low; 2: medium; and 3: high)	1–2‐3
Mature age	Age at which it can produce seeds or shoots	—
Max age	Lifespan	—
Size	The size of immature plants relative to mature plants in terms of height (1: small proportion; 2: half; 3: most; and 4: same height)	1–2–3–4
Stratum	Maximum canopy layer (height) attainable by adult individuals (1–5; from ground level to the high canopy)	1–2–3–4–5
S Disp	Short‐distance dispersal capacity	No‐Low‐Med‐High
M Disp	Medium‐distance dispersal capacity	No‐Low‐Med‐High
H Disp	Long‐distance dispersal capacity	No‐Low‐Med‐High
K Disp	Rate of decrease in dispersal curve from medium distance onward	—
Limit	Spatial extent of short, medium, and long dispersal distances in meters	—
Fecund	Number of seeds opened at a randomly selected distance each year	—
Disturbance parameters		
Age limit	Age group affected by intervention (e.g., separate for mature and immatures)	—
Seed broken	Seedling emergence rate after intervention	No‐Low‐Med‐High‐All
Propkill	Ratio of seeds and propagules killed during the intervention	No‐Few‐Half‐Most‐All
Killresp	Survival and regeneration capacity of individuals after intervention	No‐Few‐Half‐Most‐All
Respage	Functional age of shoots after intervention	—
Germination parameters		
Germination rate	Germination ability under low, medium, and high resource availability	No‐Low‐Med‐High‐All
Survival of germinants	Survival ability of seedlings under low, medium, and high resource availability	No‐Yes
Survival of immatures	Survival ability of non‐adult individuals under low, medium, and high resource availability	No‐Yes
Survival of matures	Survival ability of adult individuals under low, medium, and high resource availability	No‐Yes

**TABLE 2:** Germination and seedling survival response of functional groups included in the study for different grazing regimes. N and Y mean “No” and “Yes,” respectively, and represent the seedling survival possibility in binary form under a particular resource amount for each growth form.

Trait	Low	Medium	High	Trait	Low	Medium	High
Non‐spiny subshrub	Resource			Perennial gramineae	Resource		
Germination rate	None	Low	Medium	Germination rate	Low	Low	Low
Survival of germinants	N	N	Y	Survival of germinants	N	Y	Y
Survival of immatures	N	Y	Y	Survival of immatures	Y	Y	Y
Survival of matures	Y	Y	Y	Survival of matures	Y	Y	Y
Spiny subshrub	Resource			Annuals	Resource		
Germination rate	0 None	1 Low	2 Medium	Germination rate	1 Low	1 Low	1 Low
Survival of germinants	N	N	Y	Survival of germinants	N	N	Y
Survival of immatures	N	Y	Y	Survival of immatures	N	N	Y
Survival of matures	Y	Y	Y	Survival of matures	N	Y	Y
Perennial forb	Resource			Resprouter tree	Resource		
Germination rate	1 Low	1 Low	1 Low	Germination rate	1 Low	2 Medium	3 High
Survival of germinants	N	Y	Y	Survival of germinants	N	Y	Y
Survival of immatures	Y	Y	Y	Survival of immatures	Y	Y	Y
Survival of matures	Y	Y	Y	Survival of matures	Y	Y	Y

**TABLE 3:** Main and disturbance parameters that are assigned to functional groups in the FATELAND model in the study.

Traits	Non‐spiny subshrub	Spiny subshrub	Forb (perennial)	Gramineae (perennial)	Annuals	Resprouter tree
Main traits						
Max abundance	1	1	1	1	1	1
Mature age	2	2	1	2	1	10
Max age	30	30	5	5	2	300
Size	1	1	2	2	4	1
Stratum	1, 2	1, 2	1, 1	1, 1	1, 1	1, 3
S Disp	High	High	High	High	High	High
M Disp	Low	Low	Med	Low	Low	High
H Disp	Low	Low	Low	Low	No	High
K Disp	2, 10	2, 10	3, 20	3, 10	2, 10	2, 10
Limit (m)	5, 10, 50	5, 10, 50	5, 30, 100	5, 10, 50	5, 10, 20	10, 50, 100
Fecund	3	3	3	3	5	2
Disturbance traits						
Age limit	2, 5	2, 5	2	1	1	3, 10
Seed broken	0	0	0	0	0	0
Propkill	0	0	0	0	0	0
Killresp	(3, 3, 1) (0, 1, 2)	(3, 2, 0) (0, 1, 1)	(3, 1) (0, 2)	(3, 0) (0, 3)	(1, 3) (0, 0)	(3, 2, 0) (0, 1, 0)
Respage	(0, 1, 2)	(0, 1, 2)	(1, 2)	(1, 2)	(−1,‐1)	(1, 5, 10)

### 2.5 Model Outputs and Data Analysis

We run each scenario once due to the deterministic nature of the FATELAND model. We obtained three main outputs after running the model for 50 years of simulation. The first output includes the abundance of each functional group under each scenario and landscape for each year during the 50‐year simulation (Appendix S4). We summarized these outputs to depict the trends of abundance changes over the 50 years. The second output is the final abundance of each functional group after 50 years of simulation under each scenario (Appendix S4), visualized as mean plots for each scenario and landscape. The third is a visual representation of the vegetation structure from a bird's‐eye view, illustrating the structure of the landscape at the initial stage (before the model run) and at the final stage (after 50 years of the model run). These outputs enable us to interpret the different responses of various vegetation types in the Central Anatolian steppes (represented by initial landscapes) under various grazing regimes.

We collected raw data for a 50‐year simulation using the FATELAND model and conducted subsequent data cleaning, plotting, and analyses in the R environment (R Core Team 2023; Supplementary R code). We identified the optimal number of clusters using the Elbow method and PCA graphs using the “cluster” package (Maechler et al. 2023).

## 3 Results

**FIGURE 2:**
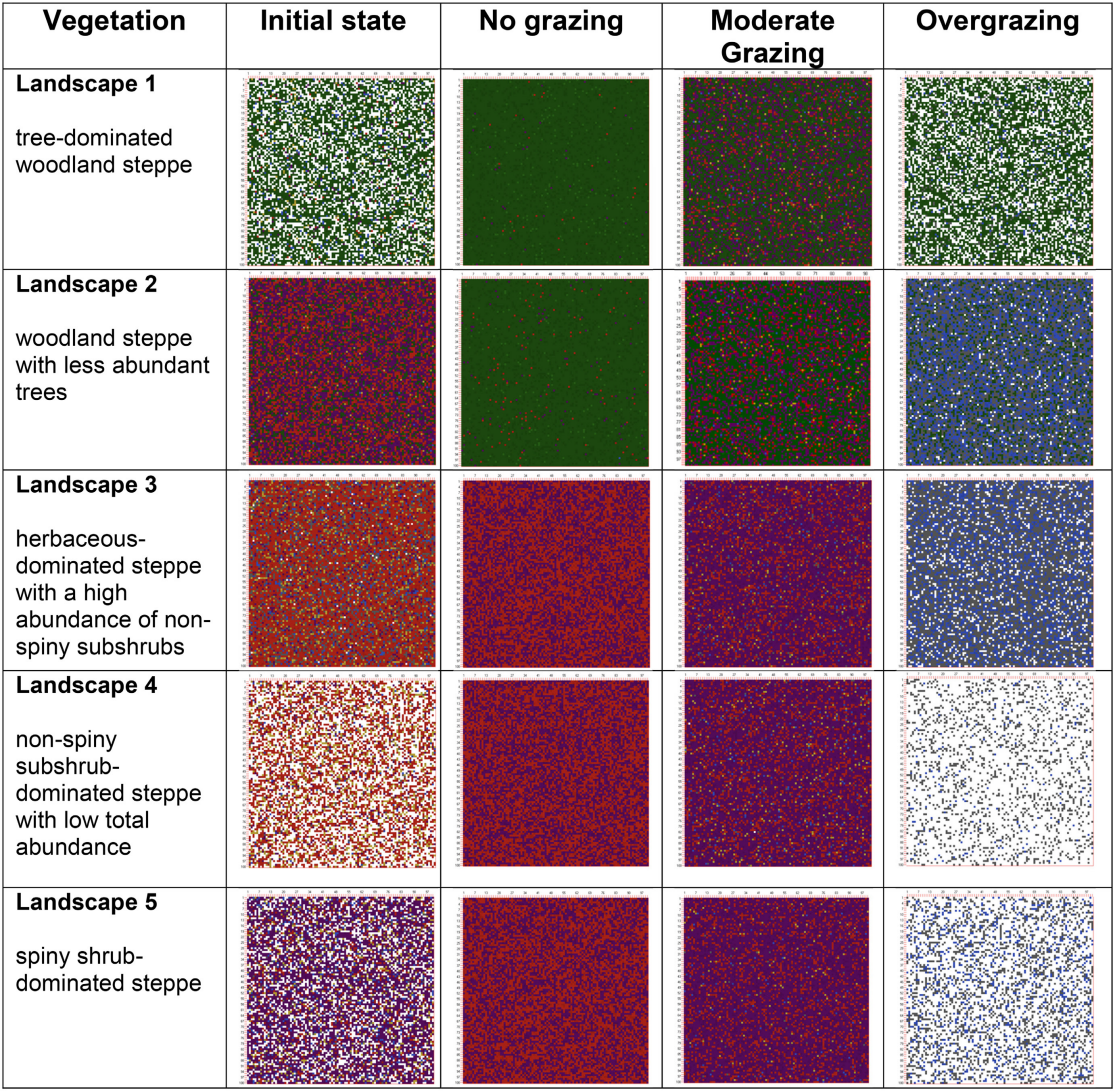
The illustration of the initial and at 50‐year final landscapes for each vegetation type under various grazing regimes. The simulated landscape consists of 10,000 cells (100 × 100 cells) covering hypothetically 1000 m × 1000 m total area in size, each representing a 10 m × 10 m area. The color in the cells indicates the dominance of a specific functional group in each cell: green for trees, red for subshrubs, purple for spiny subshrubs, orange for perennial forbs, blue for perennial gramineae, and gray for annual herbs. The white color represents empty (no vegetation) cells.

**FIGURE 3:**
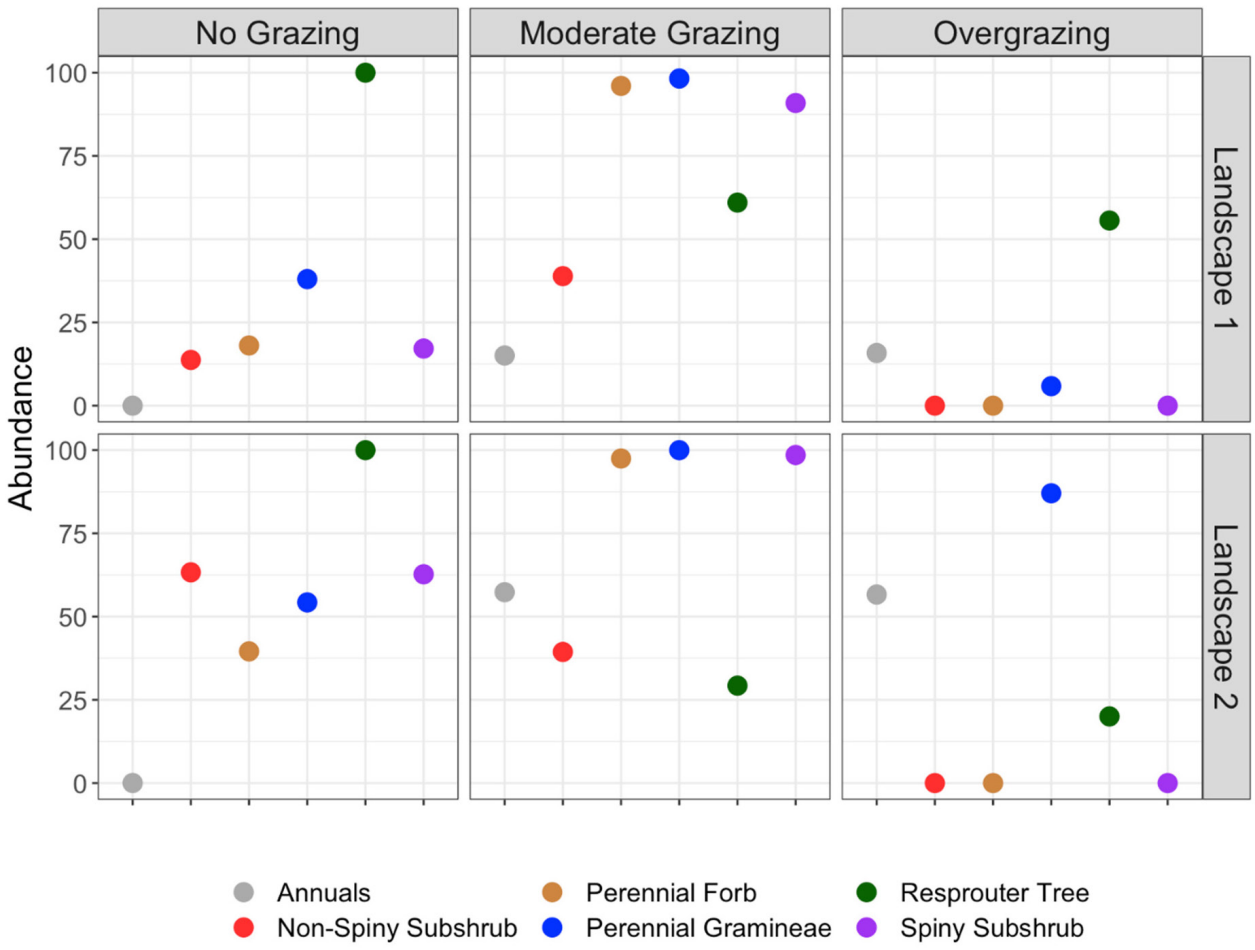
Final abundances of functional groups in the woodland steppe vegetation types (Landscapes 1 and 2) after a 50‐year simulation under various grazing regimes.

**FIGURE 4:**
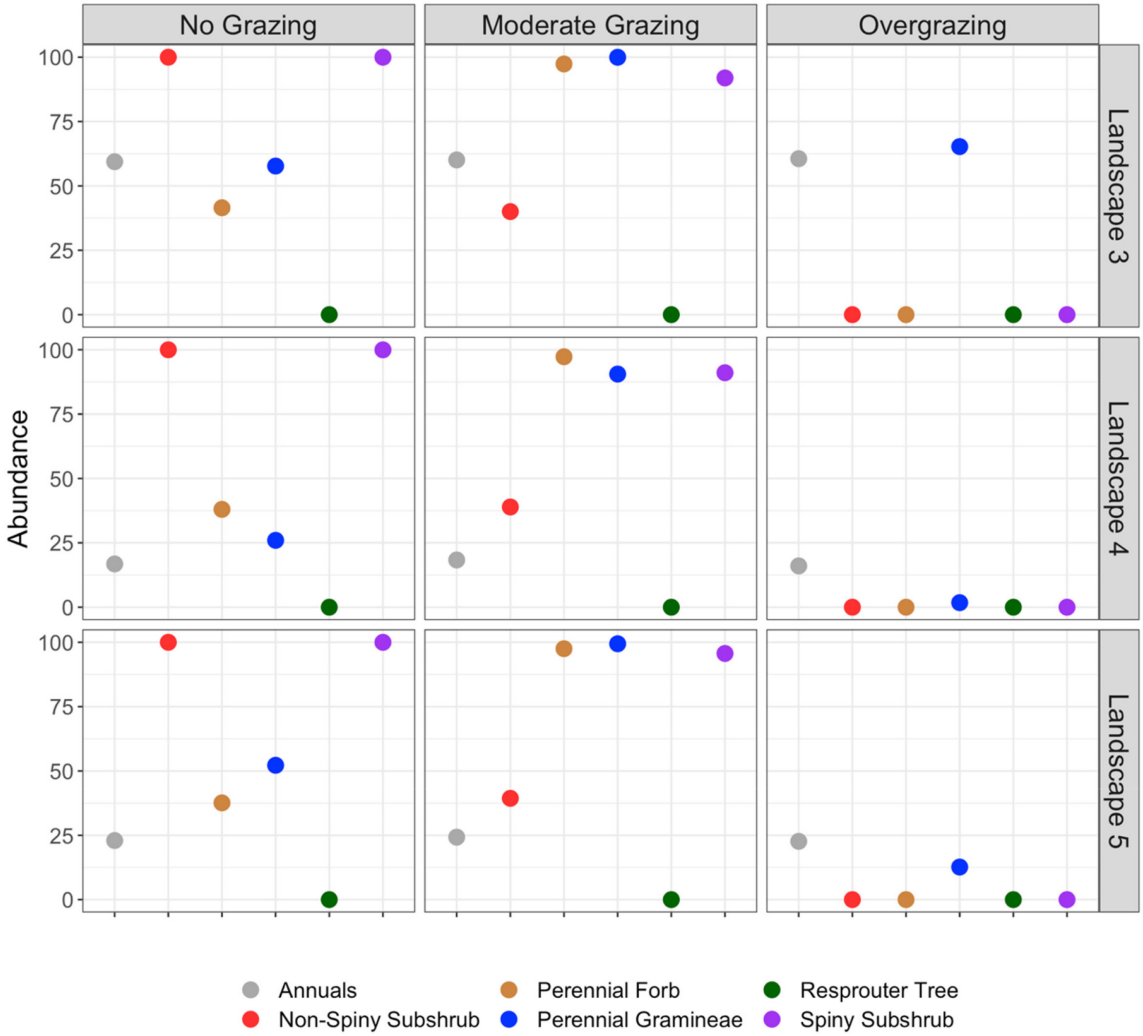
Final abundances of functional groups in the steppe (treeless) vegetation types (Landscapes 3, 4, and 5) after a 50‐year simulation under various grazing regimes.

Each landscape representing different vegetation types showed different trajectories over 50 years of simulation of vegetation dynamics under various grazing regimes. These long‐term dynamics represented vegetation state shifts in some of the simulated landscapes under specific grazing regimes, while some resulted in no considerable change in vegetation structure (Figures 2, 3, 4).

**FIGURE 5:**
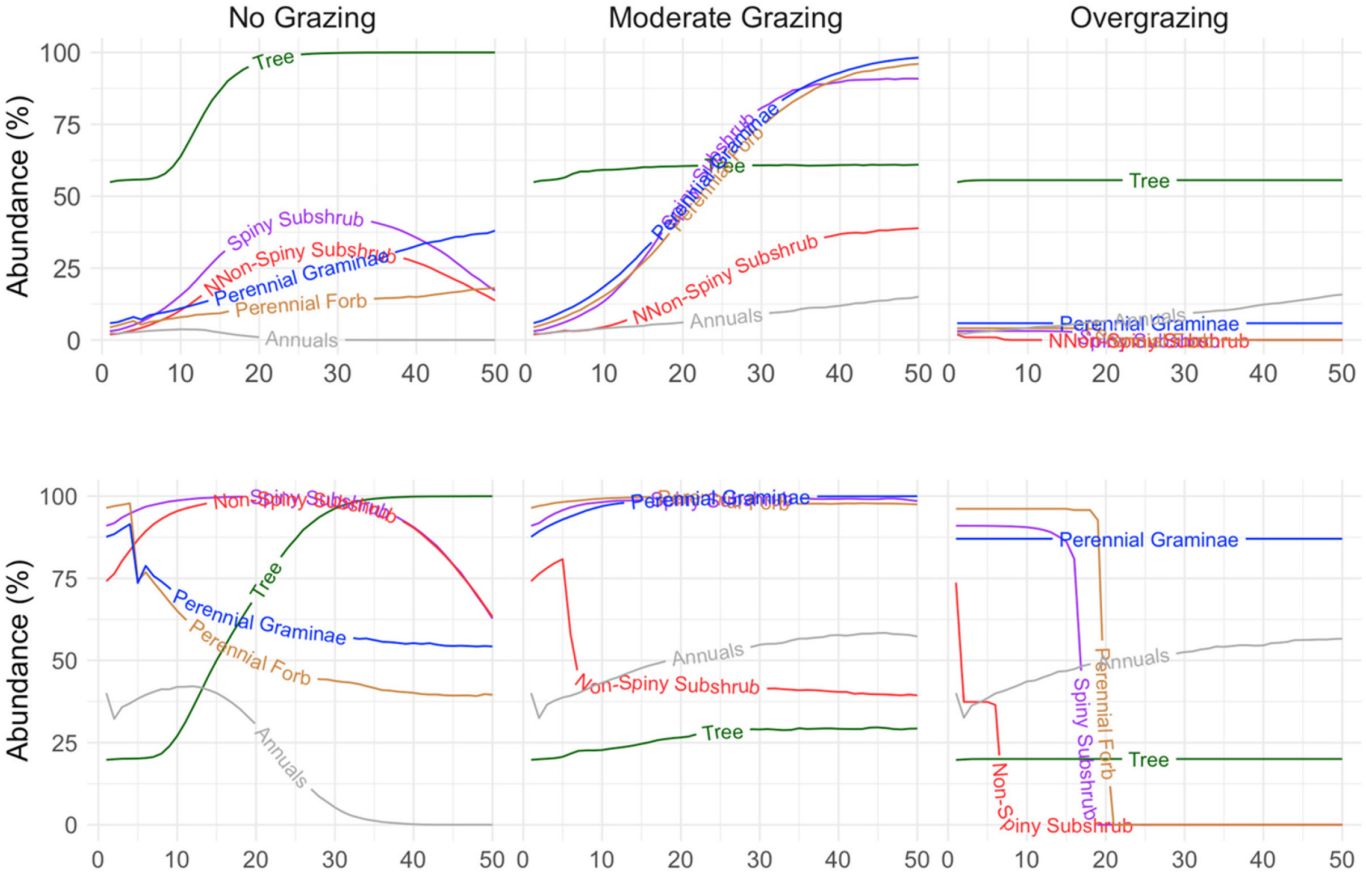
The long‐term abundance trend of functional groups in woodland steppes under varying grazing regimes over 50 years. Above panel: Landscape 1, below panel: Landscape 2. Different colors represent the abundance of different functional groups over a 50‐year period. The names of six functional groups (tree, non‐spiny subshrub, spiny subshrub, perennial gramineae, perennial forb, and annuals) are written on the curves.

In woodland steppes of Landscapes 1 and 2, the long‐term abundance of trees showed similar trends in response to specific grazing regimes with no change in moderate grazing and overgrazing regimes and a significant increase in the no‐grazing scenario (Figures 3 and 5). However, other functional groups in these two landscapes that differ in the initial abundance of these groups showed different trends in response to various grazing regimes. In Landscape 1, the abundance of these groups notably increased in response to moderate grazing, but conversely, when grazing was excluded, the increase in their abundance was suppressed by trees in the long‐term, with the exception of gramineae, which showed a slow but steady increase in abundance (Figure 5). A similar increase was also observed in Graminae, spiny subshrub, and perennial forb groups in Landscape 2 under moderate grazing regime but relatively in a less extent due to high initial abundances of these groups in this landscape (Figure 5). Notably, the abundance of non‐spiny subshrubs stabilized after a decade of decline in Landscape 2, but at the final stage had similar abundance value as in Landscape 1 under moderate grazing regime (Figures 3 and 5). In the no‐grazing scenario, the abundance of all functional groups except trees decreased in the long term in Landscape 2, too (Figures 3 and 5). Overgrazing resulted in differential responses in various functional groups in woodland steppes. Either in the case of a low (Landscape 1) or high (Landscape 2) initial abundance of subshrubs (both spiny and non‐spiny ones) and perennial forbs, these functional groups were completely lost after 50 years of overgrazing simulation (Figures 3 and 5). On the other hand, trees and gramineae kept their initial abundance and were not affected by overgrazing at all, and annual herbs showed a slight increase in their abundance over time (Figures 3 and 5).

**FIGURE 6:**
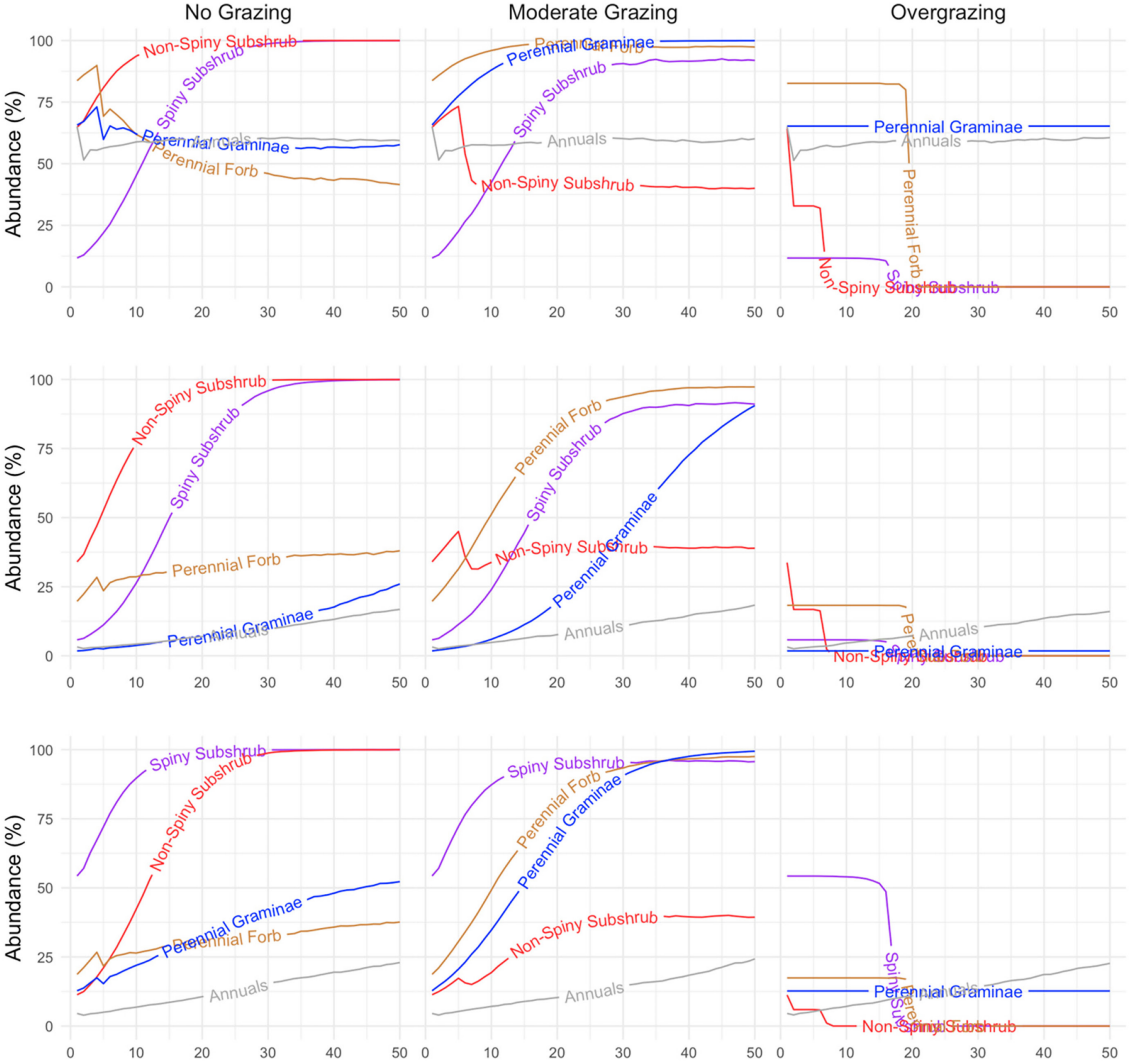
The long‐term abundance trend of functional groups in steppe vegetation types without trees under varying grazing regimes over 50 years. Above panel: Landscape 3; middle panel: Landscape 4; and below panel: Landscape 5. Different colors represent the abundance of different functional groups over a 50‐year period. The names of five functional groups (non‐spiny subshrub, spiny subshrub, perennial gramineae, perennial forb, and annuals) are written on the curves.

Different functional groups showed different trends under tested grazing regimes in treeless steppe vegetations of Landscapes 3, 4, and 5. In all these landscapes, perennial forbs, gramineae, and spiny subshrubs increased their abundance over time under the moderate grazing regime (Figure 6), resulting in their high abundance at the end of the 50 years of simulation (Figures 4 and 6). Under moderate grazing, non‐spiny subshrubs showed an increase or decrease depending on their initial abundance, ended up with a similar abundance in all three landscapes within 20 years of simulation, and their abundance remained stable until the end of the simulation (Figure 6). Grazing exclusion promoted the expansion of both spiny and non‐spiny subshrubs as their abundance significantly increases over time. Spiny subshrubs showed the same trends between no grazing and moderate grazing scenarios, but a considerable difference between these two scenarios was observed in non‐spiny shrubs, which were suppressed by moderate grazing but favored by grazing exclusion (Figure 6). In contrast, the abundance of perennial forbs and gramineae were suppressed in no grazing scenario in comparison with moderate grazing regime (Figures 4 and 6). As in landscapes representing woodland steppes, overgrazing led to the complete removal of spiny and non‐spiny subshrubs and perennial forbs, while annual herbs and gramineae maintained their initial abundance over 50 years under the overgrazing scenario in treeless steppe vegetation (Figures 4 and 6). In treeless steppe vegetation types, annual herbs showed the same trend under all three grazing regimes. In each grazing regime, the abundance of annual herbs showed a slow but steady increase when their initial abundance was relatively lower (Landscapes 4 and 5) and remained stable through time in the case of higher initial abundance (Landscape 3) (Figure 6).

## 4 Discussion

Our model results covering 50 years of simulations suggest drastic changes in vegetation structure under different grazing regimes in Central Anatolian steppes. Moderate grazing regimes promote the growth form diversity and cover of various functional groups except resprouter trees. In contrast, overgrazing and no grazing scenarios change the vegetation state in the long term. Woodland steppes tend to transform into closed woodlands under no grazing regime, while steppes without trees into dense scrubland. Overgrazing results in the total loss of subshrubs (both spiny and non‐spiny) and perennial forbs in the long term, while trees (if they exist) are not affected by overgrazing and gramineae and annual herbs promoted by overgrazing. As a result, overgrazing does not cause a vegetation state change in woodland steppes, at least within the 50‐year simulation period, but steppes without trees transform into grassland dominated by resprouter gramineae if their initial abundance is not very low. Resprouter gramineae appear to be crucial for the resilience of the plant community under overgrazing pressure, as their initial abundance remained stable across all landscape in the overgrazing scenario. On the other hand, if resprouter gramineae are absent or have low abundance in the initial vegetation, the landscape transforms into an annual herb‐dominated grassland under the overgrazing scenario.

Evidence from open ecosystems such as grasslands, savannas, and woodland steppes suggests that a lack of long‐term grazing leads to a shift toward more closed woodland vegetation (Bernardi et al. 2019; Bond 2019; Wolański et al. 2021). Our model results support this conclusion for Central Anatolian woodland steppes, showing that a lack of grazing causes a shift from open woodland steppe to closed woodland, regardless of the initial abundance of trees (whether semi‐open or open woodland). In contrast, our models also indicate that moderate grazing promotes the abundance and diversity of functional groups by maintaining the initial state of the vegetation. Enhanced diversity through moderate grazing has been observed in many herbivory‐mediated grassland ecosystems worldwide (Török et al. 2016; Joubert, Pryke, and Samways 2017; Davies et al. 2024). In savannas, moderate grazing fosters biodiversity by reducing the dominance of certain grass species, allowing less competitive species to thrive (Sankaran, Ratnam, and Hanan 2008), while overgrazing leads to a marked decline in perennial forbs and some subshrubs. Conversely, North American prairies, which have coevolved with native ungulates like bison, show a different pattern. In these ecosystems, grazing helps maintain grassland structure by preventing woody plant encroachment (Knapp et al. 1999). In the Mongolian steppes, overgrazing results in a considerable loss of biomass and diversity, especially in dry and high mountain steppes, while species richness increases under moderate grazing in more mesic steppes (Munkhzul et al. 2021). In many cases, the positive relationship between plant diversity and livestock grazing can be attributed to a long‐term history of large mammal herbivory in these ecosystems. For example, spinescence is a plant trait that serves as structural anti‐herbivore defense (Atkinson et al. 2024), and the origin of this trait can be traced back through geological history, when large herbivore mammals evolved and grazed these ecosystems (Lauenroth 1998; Charles‐Dominique et al. 2016). In our models, spinescence also emerged as a significant trait, where dominance of spiny or non‐spiny shrubs shifted notably under moderate grazing scenarios. The dominance of spiny shrubs in grazed steppe areas in Central Anatolia (Vural and Adıgüzel 2006; Kürschner and Parolly 2012; Tavşanoğlu 2017; Ülgen 2019) and other regions (Lauenroth 1998; Rahmanian et al. 2019; Atkinson et al. 2024) is frequently observed. The presence of clumps of grazing‐resistant spiny plants in steppe areas may have additional ecological importance, as they can provide refuges for many grazing‐sensitive species (Rebollo et al. 2002). Our model results, showing the dominance of spiny shrubs over non‐spiny ones under moderate grazing, suggest that the presence of spiny shrubs in a location in Central Anatolia can be used as a proxy for moderate grazing pressure, while loss of both spiny and non‐spiny shrubs can indicate overgrazing. However, it is important to note that past land use may also shape the current vegetation structure, possibly in combination with various grazing regimes. Therefore, in the Central Anatolian steppes, the response of vegetation to grazing is complex, likely influenced by the region's harsh climate, past land use, and historical grazing patterns.

In our study, overgrazing emerged as a critical driver of vegetation change in Central Anatolia, significantly reducing the abundance of all growth forms across all landscapes, except for trees in woodland steppes, which can contribute to biodiversity loss. The Eurasian steppe, specifically in Mongolia and Tibet regions, characterized by a harsher climate, exhibits even more drastic effects of grazing with rapid vegetation degradation and soil erosion when subjected to overgrazing (Wesche et al. 2016; Munkhzul et al. 2021). Our results highlight the necessity for adapted grazing management strategies that take into account the distinct ecological characteristics of the Central Anatolian steppes to prevent habitat degradation and encourage plant diversity. Consequently, the dynamics of woodland steppes highlight the resilience of trees and certain grass species under grazing pressures, which contrasts sharply with the vulnerability of perennial forbs and subshrubs. This differential response underlines the importance of understanding species‐specific traits and life histories when developing conservation strategies. For instance, the adaptability of gramineae and annual herbs to quickly regenerate makes them resilient under varying grazing intensities, suggesting that management practices need to be species and context‐specific to enhance ecosystem resilience and prevent biodiversity loss. This phenomenon aligns with the concept of disturbance‐mediated biodiversity, where moderate levels of disturbance, such as grazing, can prevent any single species from dominating an ecosystem, thereby maintaining higher species richness and ecological integrity in the plant community (Milchunas, Sala, and Lauenroth 1988; Milchunas and Lauenroth 1993). Moreover, rotational grazing systems can help quickly regenerating functional groups, increasing rangeland stability and resilience, especially in areas where grazing pressure on vegetation is expected to be high (Briske et al. 2008; de Otálora et al. 2021; Jordon et al. 2022). In this way, overgrazing can be prevented or reduced by avoiding practices that return livestock to the same area too soon and by controlling livestock numbers to ensure they do not exceed the rangeland's carrying capacity.

Under the overgrazing scenario in our models, annuals were positively or not affected as their cover increased with time especially if their abundance was low in the initial landscape. Similar results have been obtained from many studies with increasing disturbance frequency or intensity; as in increased grazing intensity transform plant communities to annual‐dominated ones in grasslands in Britain (Pakeman 2004), in high disturbance frequency in an artificial disturbance experiment in the Central Anatolian steppe (Özüdoğru, Özüdoğru, and Tavşanoğlu 2021), under heavy‐plowing treatment in regenerating pine forests in an eastern Mediterranean ecosystem (Ürker, Tavşanoğlu, and Gürkan 2018). A meta‐analysis also suggests that, on a global scale, grazing favors annual plants over perennials (Díaz et al. 2007). The life cycle and life history traits of annuals seem to be the main reasons for their resilience to high‐intensity disturbances, in our case overgrazing, as they are seeder species that are able to establish their seedlings. In overgrazing scenarios, the increase in the abundance of annuals could also be attributed to the total vanishing of other competitor functional groups, namely perennial forbs and subshrubs. However, contrasting results suggest a decrease in the proportion of annuals under overgrazing regimes in semi‐arid grasslands (Rahmanian et al. 2019). Our results also suggest that if gramineae exists in moderate abundance in the initial landscape, annuals and perennial gramineae can coexist under the overgrazing pressure. In ecosystems where herbivory pressure is managed effectively, grazing can contribute to biodiversity conservation by creating niches for a variety of plant species, thus promoting a more diverse and resilient ecosystem. For instance, in the savannas of Africa, controlled grazing has been shown to reduce the dominance of aggressive grass species, allowing for the proliferation of forbs and other grass species, which contributes to overall biodiversity (Fuhlendorf and Engle 2001; Scott‐Shaw and Morris 2015). Similarly, research in North American prairies has demonstrated that when executed with conservation goals in mind, grazing supports the maintenance of plant diversity by mimicking natural herbivory patterns that existed prior to extensive human intervention (Knapp et al. 1999). However, the relationship between grazing and biodiversity is not straightforward and depends heavily on the grazing intensity and the specific ecological context. Overgrazing, as observed in some sections of the Central Anatolian steppes, leads to significant degradation of plant communities, reducing plant cover and biodiversity (Fırıncıoğlu, Seefeldt, and Şahin 2007; Kürschner and Parolly 2012; Ambarlı et al. 2016; Rahmanian et al. 2019). This negative outcome underscores the need for implementing grazing regimes that consider ecological thresholds and are tailored to the carrying capacity of the landscape.

Results from the FATELAND model are based on simulations assuming the current climatic conditions. Therefore, we should note here that long‐term vegetation dynamics may result in different directions if the response of species to changing climate is also considered. The ongoing climatic change may increase uncertainties about the fate of vegetation dynamics under various grazing regimes, especially in the long term. While climate plays a role in shaping woodland communities in Central Anatolia (Kenar and Kikvidze 2019), the five landscapes used in our models also reflect the land use history and past grazing regimes, not just climate. Thus, our results should be interpreted in the context of different past land uses under relatively stable climatic conditions. We also did not include fire as a disturbance factor in our models, focusing solely on grazing regimes. This decision was based on the lack of wildfires in Central Anatolian grasslands over a long period due to the absence of continuous fuel, resulting from millennia of domestic grazing and agricultural activity, as well as limited knowledge of fire response in Central Anatolian steppe plants. However, it is worth to noting that the Holocene fire regimes of the region could re‐emerge due to land abandonment and climate change in the future (Tavşanoğlu 2017). Therefore, the response of Central Anatolian vegetation interactions between grazing and wildfire should be a focus of future research. Since our models are restricted to a 50‐year time period, our finding of no vegetation state change in woodland steppes under overgrazing may differ over longer periods, exceeding the lifespan of existing trees in these ecosystems. In such cases, where no new seedling establishment is expected under overgrazing, the long‐term outcome could be the eventual loss of trees and a shift in vegetation state under centuries of overgrazing.

In conclusion, our findings indicate the significant impact of grazing on the vegetation dynamics of Central Anatolian steppes and suggest the importance of adaptive management strategies that consider both the ecological characteristics of the region. Sustainable grazing practices, adjusted to the unique conditions of the Central Anatolian steppes, are essential to sustain Central Anatolian steppe vegetation states by preserving its plant functional diversity.

## Author Contributions

**Anıl Bahar:** data curation (lead), formal analysis (lead), methodology (equal), writing – original draft (lead), writing – review and editing (equal). **Çağatay Tavşanoğlu:** conceptualization (lead), formal analysis (supporting), methodology (equal), supervision (lead), writing – review and editing (equal).

## Conflicts of Interest

The authors declare no conflicts of interest.

## Supporting information


Appendix S1.

Appendix S2.

Appendix S3.

Appendix S4.

Appendix S5.

## Data Availability

The vegetation dataset, abundance‐cover values of growth forms for each landscape, raw data of model outcomes, and R codes for analyses and producing graphs are available as supplementary information. Model inputs are presented in tables within the manuscript.
