# Supplementary material for: The Effect of Grazing on Central Anatolian Steppe Vegetation: A Modeling Approach Using Functional Traits
Source: Ecol Evol. 2024 Nov 3;14(11):e70499. doi: 10.1002/ece3.70499 (PMC11532325; doi:10.1002/ece3.70499)
Supplement: Supplementary file 1 — Appendix S1. [file ECE3-14-e70499-s005.docx]

**The effect of grazing on central Anatolian steppe vegetation: A modeling approach using functional traits**

**Anıl Bahar & Çağatay Tavşanoğlu**

**Supplementary Material**

**Table S1.** Datasets and other sources used in the study for plant trait data. Source name, publication reference (if any), and the full link of data source (if any) are given.

| **Source name** | **Reference** | **Full link** |
| --- | --- | --- |
| TRY Plant Trait Database | Kattge et al. (2020) | try-db.org |
| BROT Plant Trait Database | Tavşanoğlu & Pausas (2018) | figshare.com/collections/BROT_2_0_A_functional_trait_database_for_Mediterranean_Basin_plants/3843841 |
| The Flora of Turkey and east Aegean Islands | Davis (1965-1985) | - |
| World Flora Online | Borsch (2020) | worldfloraonline.org/ |
| TÜBİVES | Bakış (2011) | tubives.com/ |
| Missouri Botanical Garden | - | missouribotanicalgarden.org |
| The PLANTS Database | USDA (2024) | plants.usda.gov |
| BGCI Plant Search | BGCI (2024) | plantsearch.bgci.org |
| Flora Helvetica | - | flora-helvetica.ch |
| National Digital Herbarium Database | - | herbaryum.tagem.gov.tr |
| The Virtual Herbarium of Lake Van Basin | - | vanherbaryum.yyu.edu.tr |
| Flora Anatolica | - | floranatolica.com |

**References**

Bakış, Y., Babaç, M. T., & Uslu, E. (2011). Updates and improvements of Turkish Plants Data Service (TÜBİVES). In 6th International Symposium on Health Informatics and Bioinformatics (HIBIT) (pp. 136-140).

BGCI (2024). PlantSearch. Botanic Gardens Conservation International. Richmond, U.K.

Borsch, T., Berendsohn, W., Dalcin, E. et al. (2020). World Flora Online: Placing Taxonomists at the Heart of a Definitive and Comprehensive Global Resource on the World’s Plants, *Taxon*, 69, 1311–1341.

Davis, P.H. (ed.) (1965–1985). *Flora of Turkey and the East Aegean Islands*. Vols. 1–9. Edinburgh University Press, Edinburgh.

Kattge, J. et al. (2020). TRY plant trait database – enhanced coverage and open access. *Global Change Biology*, 26,119-188.

Tavşanoğlu, Ç., & Pausas, J. G. (2018). A functional trait database for Mediterranean Basin plants. *Scientific Data*, 5, 180135.

USDA, NRCS. 2024. The PLANTS Database (http://plants.usda.gov). National Plant Data Team, Greensboro, NC USA.

| 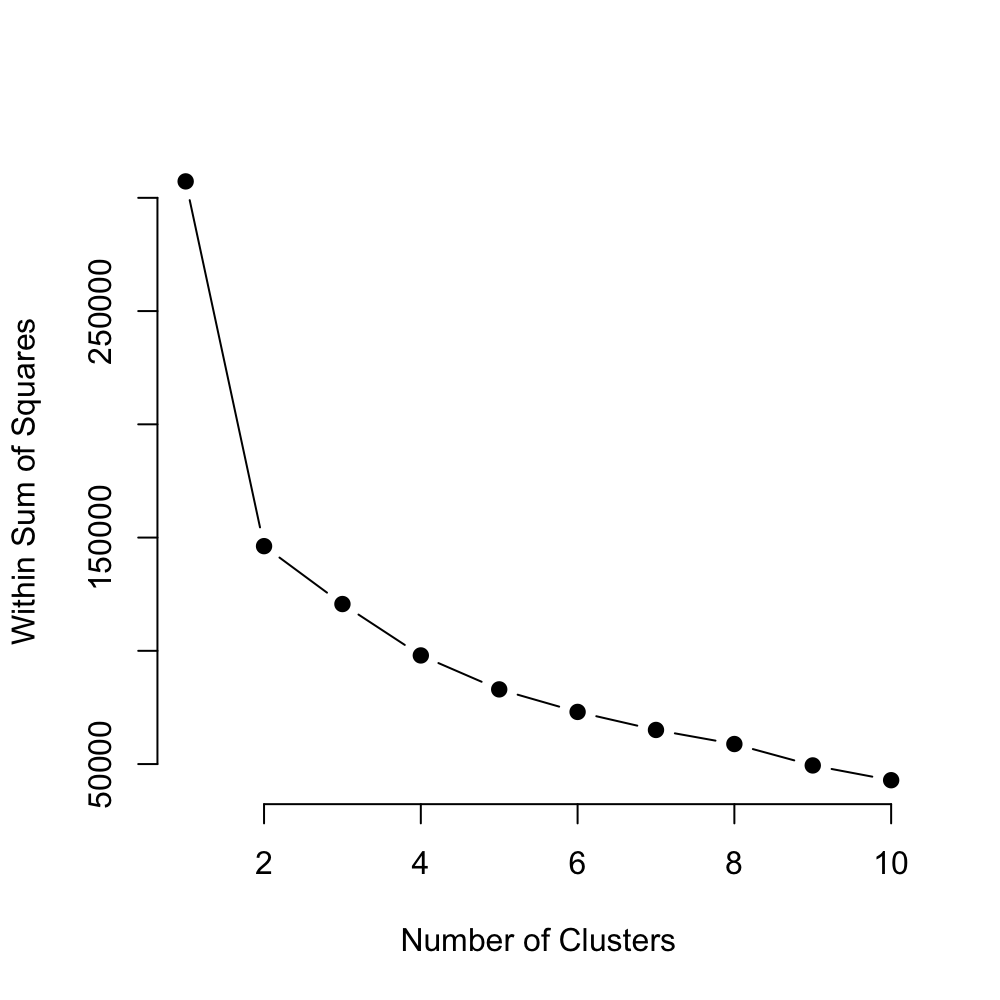 | 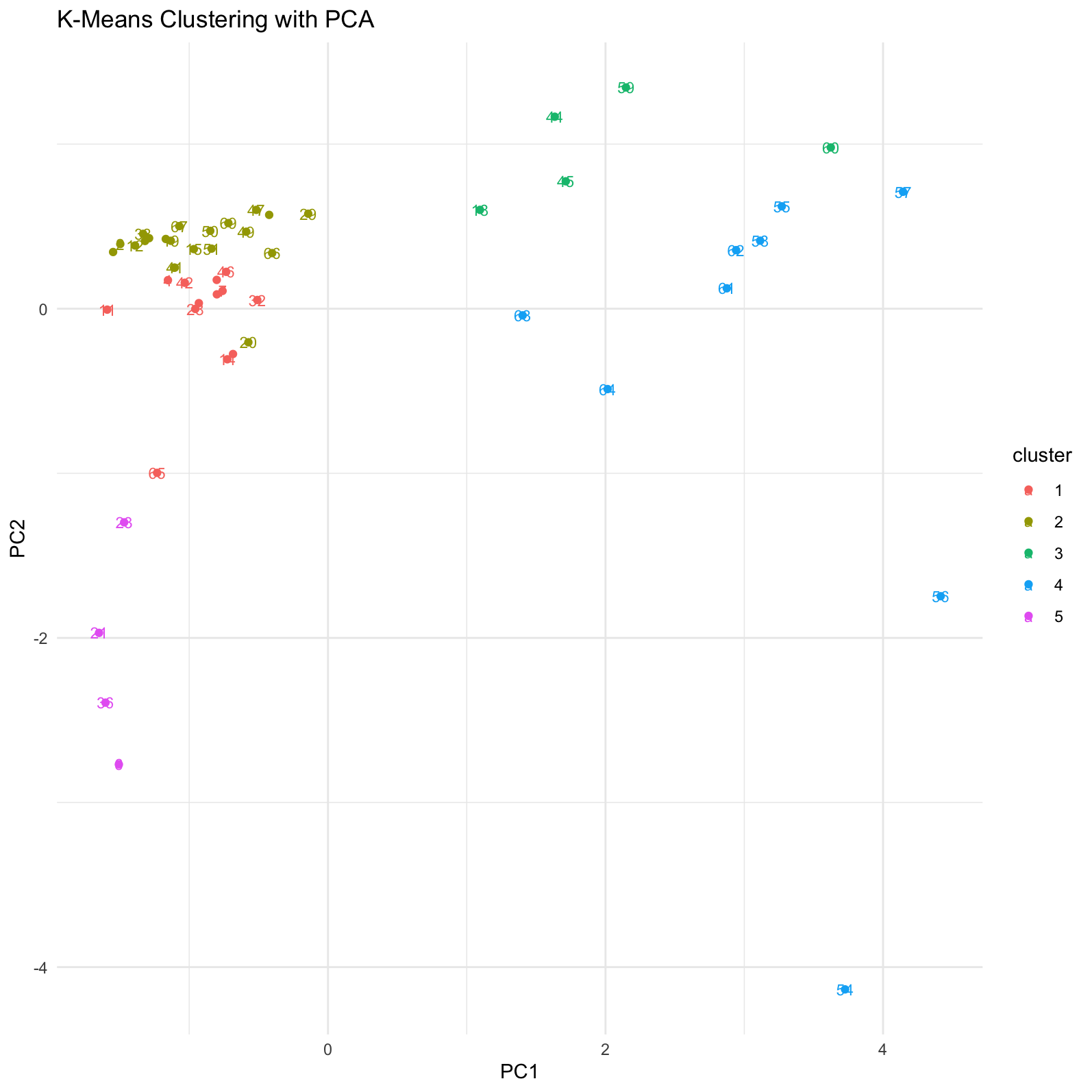 |
| --- | --- |

**Figure S1.** The graph for determining the cluster number with the Elbow method (left panel) and visualization of five selected clusters in a two-dimensional plane using k-means clustering and principal component analysis (right panel).
